# Pterostilbene Induces Apoptosis from Endoplasmic Reticulum Stress Synergistically with Anticancer Drugs That Deposit Iron in Mitochondria

**DOI:** 10.3390/ijms25052611

**Published:** 2024-02-23

**Authors:** Yukiko Nishiguch, Rina Fujiwara-Tani, Shota Nukaga, Ryoichi Nishida, Ayaka Ikemoto, Rika Sasaki, Shiori Mori, Ruiko Ogata, Shingo Kishi, Yudai Hojo, Hisashi Shinohara, Masayuki Sho, Hiroki Kuniyasu

**Affiliations:** 1 Department of Molecular Pathology, Nara Medical University, 840 Shijo-cho, Kashihara 634-8521, Nara, Japan; yukko10219102@yahoo.co.jp (Y.N.); shota.nukaga@gmail.com (S.N.); g.m__r1@outlook.jp (R.N.); a.ikemoto.0916@gmail.com (A.I.); rika0st1113v726296v@icloud.com (R.S.); m.0310.s.h5@gmail.com (S.M.); pkuma.og824@gmail.com (R.O.); nmu6429@yahoo.co.jp (S.K.); 2 Pathology Laboratory, Research Institute, Tokushukai Nozaki Hospital, 2-10-50 Tanigawa, Daito 574-0074, Osaka, Japan; 3 Department of Surgery, Hyogo College of Medicine, 1-1 Mukogawa-cho, Nishinomiya 663-8501, Hyogo, Japan; yudaihojo@outlook.com (Y.H.); shinohara@hyo-med.ac.jp (H.S.); 4 Department of Surgery, Nara Medical University, Kashihara 634-8522, Nara, Japan; m-sho@naramed-u.ac.jp

**Keywords:** pterostilbene, PDZD8, mitochondria, MAM, ER stress

## Abstract

Anticancer agents are playing an increasing role in the treatment of gastric cancer (GC); however, novel anticancer agents have not been fully developed. Therefore, it is important to investigate compounds that improve sensitivity to the existing anticancer drugs. We have reported that pterostilbene (PTE), a plant stilbene, enhances the antitumor effect of low doses of sunitinib in gastric cancer cells accumulating mitochondrial iron (II) (mtFe) at low doses. In this study, we investigated the relationship between the mtFe deposition and the synergistic effect of PTE and different anticancer drugs. For this study, we used 5-fluorouracil (5FU), cisplatin (CPPD), and lapatinib (LAP), which are frequently used in the treatment of GC, and doxorubicin (DOX), which is known to deposit mtFe. A combination of low-dose PTE and these drugs suppressed the expression of PDZ domain-containing 8 (PDZD8) and increased mtFe accumulation and mitochondrial H_2_O_2_. Consequently, reactive oxygen species-associated hypoxia inducible factor-1α activation induced endoplasmic reticulum stress and led to apoptosis, but not ferroptosis. In contrast, 5FU and CDDP did not show the same changes as those observed with PTE and DOX or LAP, and there was no synergistic effect with PTE. These results indicate that the combination of PTE with iron-accumulating anticancer drugs exhibits a strong synergistic effect. These findings would help in developing novel therapeutic strategies for GC. However, further clinical investigations are required.

## 1. Introduction

Gastric cancer (GC) is currently the third and second leading cause of cancer-related death in Japan and worldwide, respectively [1,2]. Although the overall 5-year survival rate for this disease is 73.1% [3], the prognosis of patients with advanced GC remains poor, with the 5-year survival rate for stage IV cases being only 7.3% [3]. Recently, the benefits of using multidisciplinary treatment including major chemotherapeutic agents, such as cisplatin (CDDP), 5-fluorouracil (5FU), and taxanes, for such advanced cases have been highlighted [4]. Combination therapy in cancer treatment is the use of different drugs target cancer cells through multiple mechanisms. This approach offers several advantages in the management of cancer. Increased therapeutic efficacy, reduced drug resistance, synergistic effects, minimized toxicity, overcoming target heterogeneity, and optimization of personalized therapy are some of the benefits of combination therapy. Therefore, the development of new anticancer drugs and new combination therapies will play an important role in cancer treatment [5].

The currently recommended molecular targeted therapy for GC is trastuzumab, which targets human epidermal growth factor receptor 2 (HER2) [4]. The survival rate of patients with HER2-positive GC is 22%, and trastuzumab can extend their survival [6]. However, the frequency of HER2-positive GC is less than 20% [7]. Therefore, there is a need for new molecular targeted therapies and for developing novel methods to sensitize tumors to the existing anticancer drugs.

Pterostilbene (PTE) is an abundant dietary nutrient in blueberries [8]. PTE inhibits cancer cell proliferation in a concentration-dependent manner, lowers mitochondrial membrane potential, and induces cell apoptosis [9,10]. We previously reported that PTE suppresses cancer stem cell activity [11]. Additionally, PTE shows synergistic antitumor effects against GC cell lines when used in combination with sunitinib (SUN) [12]. SUN suppresses the expression of PDZ domain-containing 8 (PDZD8), resulting in the deposition of mitochondrial iron (II) (mtFe). Iron influences cancer cell survival and proliferation through a variety of cellular processes: cell proliferation, energy metabolism, angiogenesis, oxidative stress, and immune response [13]. In particular, mitochondrial iron, as an iron–sulfur complex, is essential for energy production and other processes, while it is involved in cell death through ferroptosis [14]. In this work, the significance of mitochondrial iron deposition was also studied.

In this study, we aimed to investigate the synergistic effects of the combination of PTE with 5FU, CDDP, and LAP, which are frequently used to treat GC, and doxorubicin (DOX), which is known to affect mtFe deposition [15], on mtFe deposition in GC.

## 2. Results

### 2.1. Effect of High-Dose PTE on GC Cell Lines

**Figure 1:**
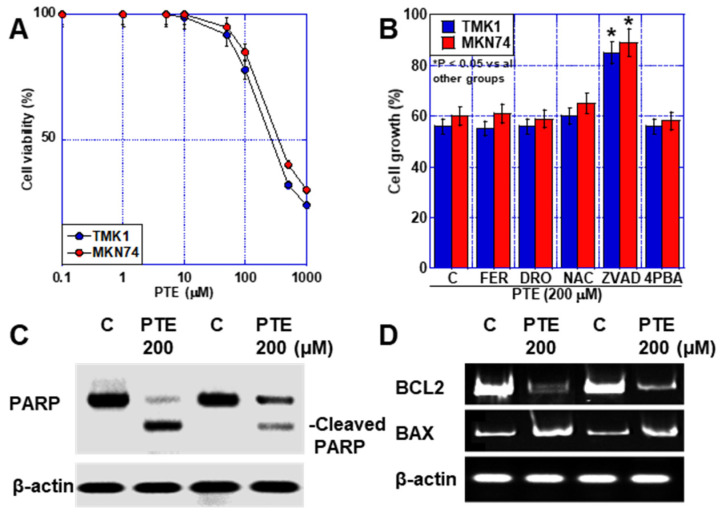
Effect of PTE-H in GC cells. (**A**) Effect of PTE on cell growth in GC cell lines, TMK1, and MKN74. (**B**) Cell death inhibitor assay. Cells were treated with PTE-H (200 μM) for 48 h with or without cell inhibitors. * *p* < 0.05 vs. all other groups. (**C**) PARP cleavage by PTE-H. (**D**) Effect of PTE-H on expression of *BCL2* and *BAX*. Error bar, standard deviation from three independent trials. * Significance was calculated using Tukey methods. GC, gastric cancer; PTE, pterostilbene; PTE-H, high-dose PTE; *BCL2*, B-cell lymphoma 2 gene; *BAX*, BCL-2-associated X protein gene; C, control; FER, ferrostatine-1; DFO, deferoxamine; NAC, N-acetyl-L-cysteine; ZVAD, Z-VAD-FMK; 4PBA, 4-phenylbutyric acid; PARP, poly ADP-ribose polymerase.

First, we investigated the effect of PTE on GC cell lines (Figure 1A). A concentration-dependent inhibition of cell proliferation was observed. Thereafter, we conducted studies separately for high-dose PTE (PTE-H, 200 μM) and low-dose PTE (PTE-L, 10 μM). PTE-H inhibited the growth of both TMK1 and MKN74 GC cells by approximately 40%. PTE-H-induced cell death was investigated using various cell death inhibitors (Figure 1B), including ZVAD (apoptosis), ferrostatin (FER), deferoxamine (DFO) (ferroptosis), N-acetylcysteine (NAC, antioxidant), and 4-phenylbutyric acid (4PBA, endoplasmic reticulum [ER] stress). Cell death inhibitor assays showed that cell death was rescued by ZVAC, but not by FER, DFO, NAC, or 4PBA. In PTE-H cells, poly[ADP-ribose] polymerase (PARP) cleavage was observed, confirming apoptosis (Figure 1C). In terms of apoptosis-related gene expression, PTE-H treatment decreased *BCL2* expression and increased *BAX* expression (Figure 1D).

### 2.2. Relationship of Accumulation of mtFe with Anticancer Drugs and Sensitizing Effects of PTE

**Figure 2:**
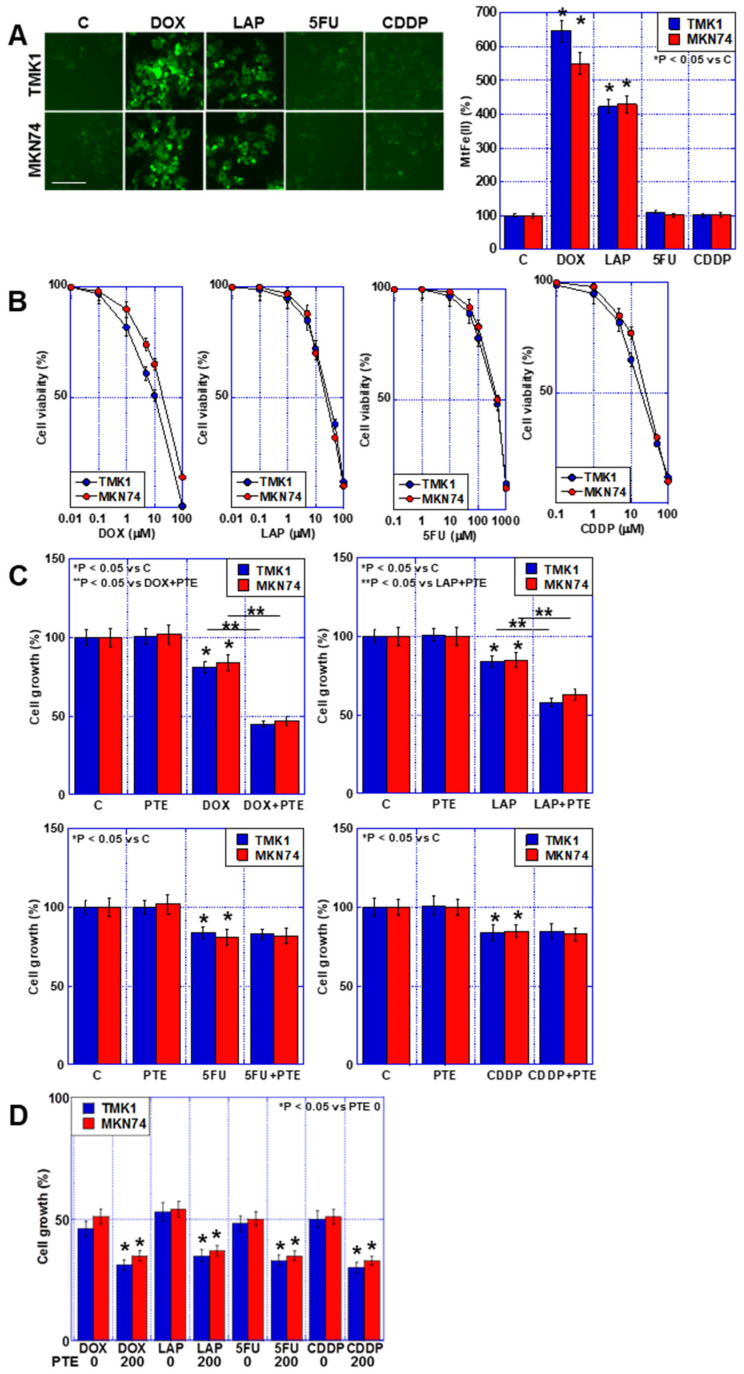
Accumulation of mtFe and PTE-L in GC cells. (**A**) Accumulation of mtFe in GC cells treated with PTE-L (10 μM) for 48 h. Scale bar, 50 µm. (Right) Semi-quantification of mtFe. * *p* < 0.05 vs. C. (**B**) Sensitivity to anti-cancer drugs: DOX, LAP, 5FU, CDDP. (**C**) Effect of concurrent treatment with anti-cancer drugs (IC_20_) and PTE-L. * *p* < 0.05 vs. C, *p* < 0.05 vs. DOX+PTE, or ** *p* < 0.05 vs. LAP+PTE. (**D**) Effect of concurrent treatment with anti-cancer drugs (IC_20_) and PTE-H (200 μM). * *p* < 0.05 vs. PTE 0. Error bar, standard deviation from three independent trials. *^,^** Significance was calculated using ANOVA. GC, gastric cancer; PTE, pterostilbene; PTE-H, high-dose PTE; PTE-L, low-dose PTE; C., control; mtFe, mitochondrial iron (II); DOX, doxorubicin; LAP, lapatinib; 5FU, 5-fluorouracil; CDDP, cisplatin; IC, inhibitory concentration.

In a previous study, an increased synergistic antitumor effect was observed between SUN and PTE, which involved mtFe deposition [11]. Therefore, we investigated the effects of mtFe deposition on anticancer drugs using DOX, LAP, 5FU, and CDDP (Figure 2A). DOX and LAP increased mtFe, whereas 5FU and CDDP did not. 

Next, we investigated the growth-inhibiting effects of the four anticancer drugs on the human GC cell lines TMK1 and MKN74 (Figure 2B). A concentration-dependent inhibition of cell proliferation was observed in both cell lines after treatment with each individual anticancer drug. In subsequent studies, a concentration equivalent to IC_20_ was used. Next, we investigated the combined effects of the four anticancer drugs at IC_20_ and a low concentration of PTE (PTE-L, 10 μM) (Figure 2C). In all GC cells, the combination of PTE-L and DOX or LAP exhibited a synergistic inhibitory effect on cell proliferation, whereas no additive inhibitory effect was observed with 5FU and CDDP. In contrast, a combination of PTE-H (200 μM) and the four anticancer drugs at IC_20_ showed additive growth inhibition in all cells (Figure 2D). 

### 2.3. Effect of Combined Use of PTE-L and mtFe-Depositing Anticancer Drugs on Mitochondria

**Figure 3:**
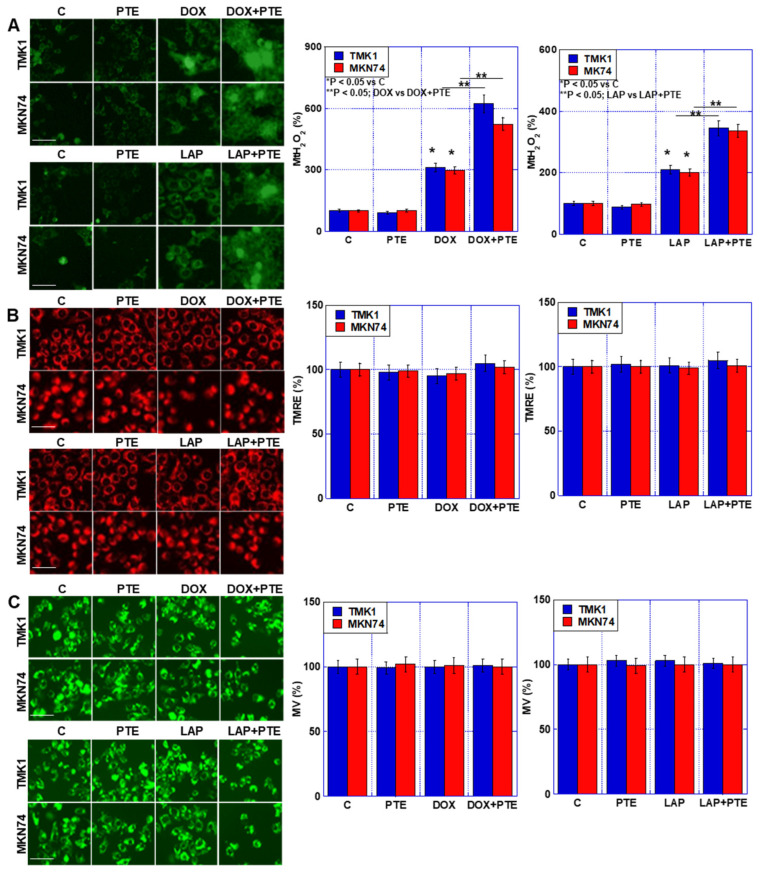
Effect of concurrent treatment with PTE-L and DOX or LAP. GC cells were treated with PTE-L (10 μM) and DOX or LAP (IC_20_) for 48 h. (**A**) MtH_2_O_2_, (**B**) mitochondrial membrane potential determined based on TMRE. (**C**) MV based on mitogreen (right) semi-quantification of the fluorescence intensity. Scale bar, 50 µm. * *p* < 0.05 vs. C, ** *p* < 0.05; DOX vs. DOX+PTE, or ** *p* < 0.05; LAP vs. LAP+PTE. *^,^** Significance was calculated using ANOVA with Bonferroni correction. Error bar, standard deviation from three independent trials. GC, gastric cancer; PTE, pterostilbene; PTE-H, high-dose PTE; PTE-L, low-dose PTE; C, control; DOX, doxorubicin; LAP, lapatinib; IC, inhibitory concentration; MtH_2_O_2_, mitochondrial H_2_O_2_; TMRE, tetramethylrhodamine ethyl ester; MV, mitochondrial volume.

To investigate the effects of the combined use of PTE-L, DOX, and LAP on mitochondria, TMK1 and MKN74 GC cells were treated (Figure 3). Increased H_2_O_2_ levels were observed in both cell lines (Figure 3A). In contrast, no changes were observed in mitochondrial membrane potential or mitochondrial mass, even when PTE-L and anticancer drugs were combined (Figure 3B,C).

### 2.4. Cell Death Due to the Combination of PTE-L and mtFe-Depositing Anticancer Drugs

**Figure 4:**
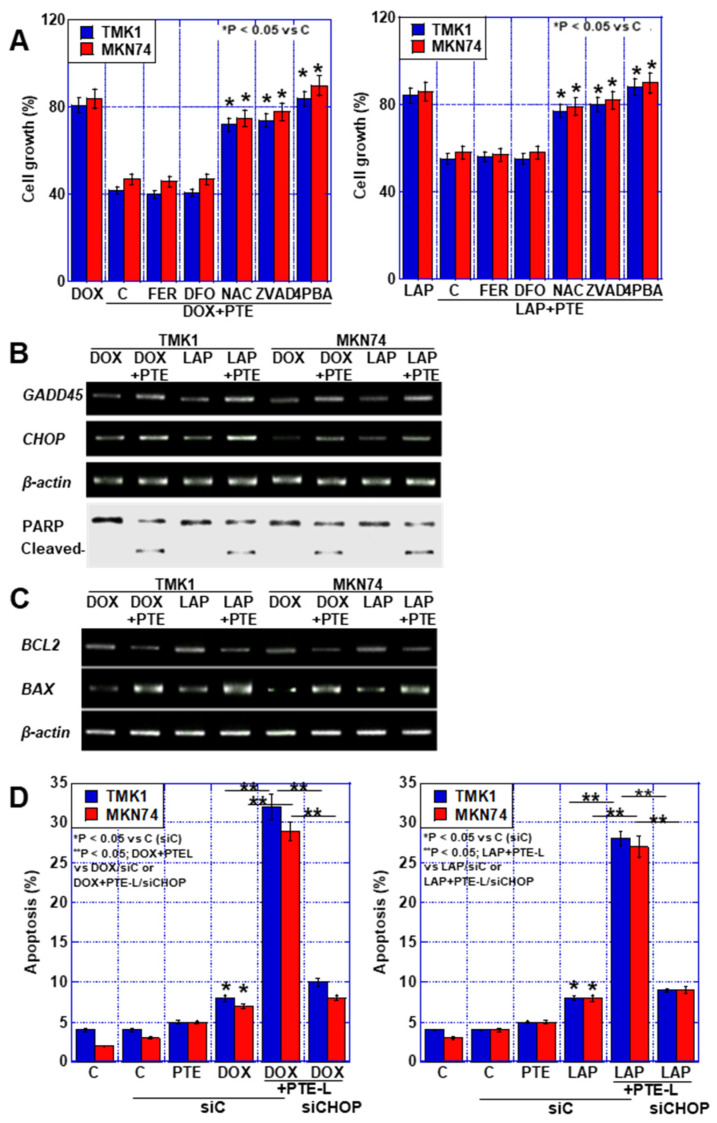
Cell death in GC cells treated with a combination of PTE-L and DOX or LAP. (**A**) Cell death inhibitor assay in GC cells exposed to concurrent treatment with PTE-L and DOX (IC20) (left) or PTE-L and LAP (IC 20) (right) * *p* < 0.05 vs. C. (**B**) ER stress-associated gene expression and PARP cleavage. (**C**) Expression of *BCL2* and *BAX*. (**D**) Effect of *CHOP* knockdown on cell death caused by concurrent treatment with PTE-L and DOX (left) or LAP (right). * *p* < 0.05 vs. C (siC), ** *p* < 0.05; DOX+PTEL vs. DOX/siC or DOX+PTE-L/siCHOP (left), or ** *p* < 0.05; LAP+PTE-L vs. LAP/siC or LAP+PTE-L/siCHOP (right). Error bar, standard deviation from three independent trials. *^,^** Significance was calculated using ANOVA with Bonferroni correction.

As mentioned above, a synergistic effect of low-concentration PTE on DOX and LAP, which have mtFe deposition effects, was observed. Therefore, we performed a cell death inhibitor assay on cells treated with these drugs in combination with PTE-L (Figure 4A). The synergistic effect of DOX or LAP at IC_20_ with PTE-L on cell death was not restored by FER or DFO. In contrast, approximately 90% recovery was observed with ZVAD and NAC, and complete recovery was observed with 4-PBA, the ER stress inhibitor.

GC, gastric cancer; PTE, pterostilbene; PTE-L, low-dose PTE; C, control; DOX, doxorubicin; LAP, lapatinib; IC, inhibitory concentration; ER, endoplasmic reticulum; GADD45, growth arrest and DNA damage inducible 45; CHOP, CCAAT/enhancer-binding protein homologous protein; PARP, poly ADP-ribose polymerase; *BCL2*, B cell lymphoma 2; *BAX*, BCL-2-associated X protein; C, control; FER, ferrostatine-1; DFO, deferoxamine; NAC, N-acetyl-L-cysteine; ZVAD, Z-VAD-FMK; 4PBA, 4-phenylbutyric acid; siC, control small interfering RNA; siCHOP, small interfering RNA for *CHOP*.

Consequently, we investigated the expression of growth arrest and DNA damage-inducible (*GADD45*) and CCAAT/enhancer-binding protein homologous protein (*CHOP*) genes to confirm that the synergistic effect was due to ER stress (Figure 4B). The expression of both genes was increased by the combination of anticancer drugs and PTE-L. 

Furthermore, PARP cleavage was not observed with DOX or LAP (IC_20_) alone, but was observed when these drugs were combined with PTE-L. This combination treatment decreased *BCL2* expression and increased *BAX* expression (Figure 4C). In contrast, *CHOP*-knockdown suppressed the cell death caused by the combination of DOX or LAP and PTE-L (Figure 4D). These results suggested that the combination of PTE-L and mtFe-depositing anticancer drugs induced apoptosis in cancer cells in response to ER stress. 

### 2.5. Synergistic Effect of PTE and Anticancer Drugs on mtFe Deposition

**Figure 5:**
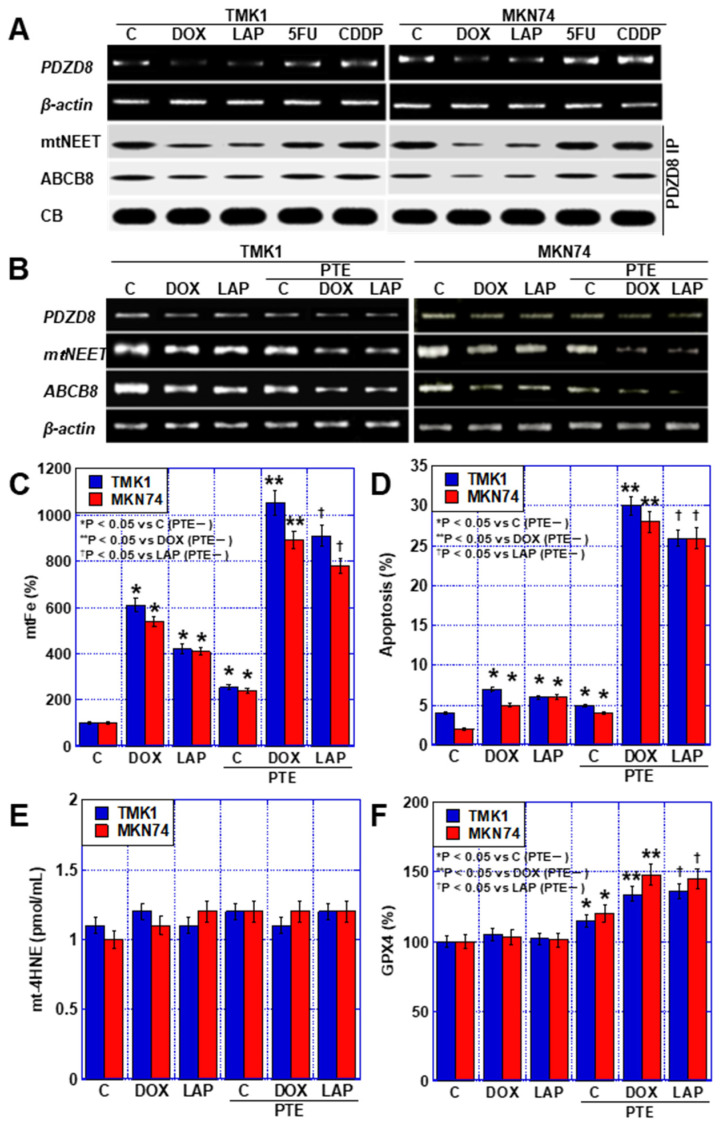
MAM in GC cells treated with PTE-L and DOX or LAP. (**A**) Expression of *PDZD8* and PDZD8-associated mtNEET and ABCB8. (**B**) Expression of MAM-associated genes. (**C**–**F**) Effect of concurrent treatment with PTE-L and DOX or LAP (IC_20_) on mtFe, (**C**), apoptosis (**D**), mt-HNE4, (**E**) and mitochondrial GPX4 expression (**F**). * *p* < 0.05 vs. C (PTE−), ** *p* < 0.05 vs. DOX (PTE−), ^†^ *p* < 0.05 vs. LAP (PTE−). Error bar, standard deviation from three independent trials. *^,^**^,†^ Significance was calculated using ANOVA with Bonferroni correction. GC, gastric cancer; PTE, pterostilbene; PTE-L, low-dose PTE; C, control; DOX, doxorubicin; LAP, lapatinib; 5FU, 5-fluorouracil; CDDP, cisplatin; IC, inhibitory concentration; C, con; PDZD8, PDZ domain-containing 8; mtNEET, mitochondrial protein containing Asn–Glu–Glu–Thr (NEET) sequence; ABCB8, ATP-binding cassette subfamily B member 8; MAM, mitochondria-associated endoplasmic reticulum membrane; CB, Coomassie blue; IP, immunoprecipitation; mtFe, mitochondrial iron (II); mt-HNE4, mitochondrial hydroxynonenal-4; GPX4, glutathione peroxidase 4.

Next, the mechanism of mtFe deposition in GC cells by DOX and LAP was examined. We previously reported that PTE reduces PDZD8, a protein in MAM [11]. Therefore, we investigated the effects of the four anticancer drugs on PDZD8 expression (Figure 5A). PDZD8 expression was decreased by DOX and LAP (mtFe-depositing drugs), whereas no change was observed with 5FU and CDDP (mtFe-non-depositing drugs). Next, we investigated the expression of mitochondrial protein containing the Asn-Glu-Glu-Thr (NEET) sequence (mitoNEET; mtNEET) and that of the ATP-binding cassette subfamily B member 8 (ABCB8), which bind to PDZD8 and are involved in iron transport. In both cases, the levels of PDZD8-binding proteins decreased with the DOX and LAP treatment, whereas no changes were observed with the 5FU and CDDP treatment (Figure 5A). Furthermore, we examined changes in the mRNA levels of *PDZD8*, *mtNEET*, and *ABCB8* when DOX and LAP were used alone or in combination with PTE-L (Figure 5B). The expression of *PDZD8*, *mtNEET*, and *ABCB8* was decreased by treatment with DOX, LAP, and PTE-L alone, and was further decreased when each drug was combined with PTE-L. mtFe accumulation and apoptosis were increased by treatment with DOX, LAP, and PTE-L alone and were further increased when each drug was combined with PTE-L (Figure 5C,D). In contrast, mitochondrial lipid peroxidation (mt-4HNE) did not change with treatment with DOX, LAP, or PTE-L alone, or when the drugs were combined with PTE-L (Figure 5E). The expression of glutathione peroxidase (GPX)-4 protein, which plays an important role in mitochondrial redox, was increased after treatment with DOX, LAP, and PTE-L alone, and was further increased when each drug was combined with PTE-L (Figure 5F). 

**Figure 6:**
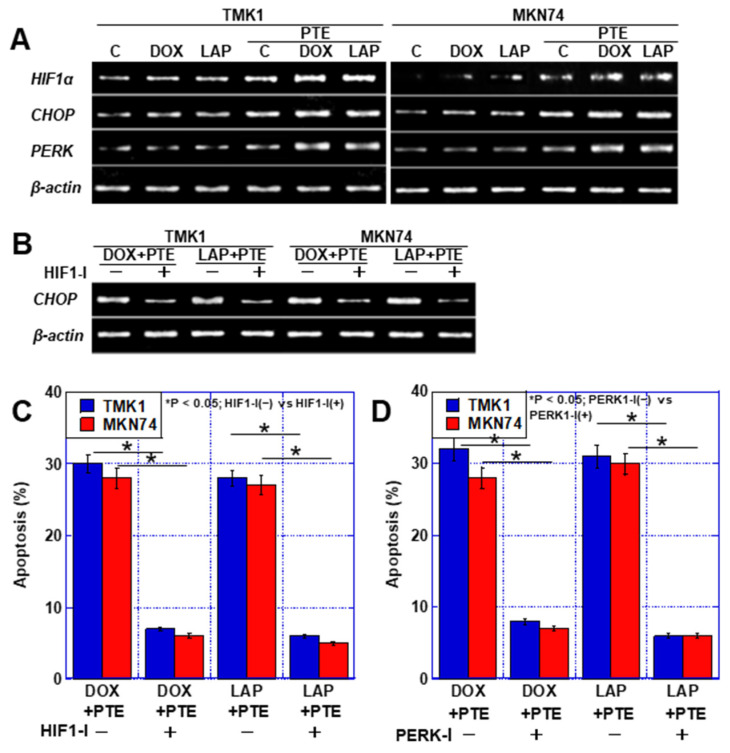
ER stress in GC cells treated with PTE-L and DOX or LAP. (**A**) Expression of ER stress-associated genes. (**B**,**C**) Effect of HIF1α inhibitor on *CHOP* expression (**B**) and apoptosis (**C**). * *p* < 0.05; HIF1-I(−) vs. HIF1-I(+). (**D**) Effect of PERK inhibitor on apoptosis. * *p* < 0.05; PERK1-I(−) vs. PERK1-I(+). Error bar, standard deviation from three independent trials. * Significance was calculated using ANOVA with Bonferroni correction. GC, gastric cancer; PTE, pterostilbene; PTE-L, low-dose PTE; C, control; DOX, doxorubicin; LAP, lapatinib; IC, inhibitory concentration; C, con; PDZD8, PDZ domain-containing 8; mtNEET, mitochondrial protein containing Asn–Glu–Glu–Thr (NEET) sequence; ABCB8, ATP-binding cassette subfamily B member 8; HIF, hypoxia-inducible factor; mtFe, mitochondrial iron (II); mt-HNE4, mitochondrial hydroxynonenal-4; GPX4, glutathione peroxidase 4.

Next, factors linking endoplasmic reticulum stress and apoptosis were examined. No obvious changes were observed in the RNA expression of hypoxia-inducible factor-1α (*HIF1α*) and *CHOP* after treatment with DOX, LAP, and PTE-L alone, but their expression levels increased upon treatment with the combination of each drug with PTE-L (Figure 6A). The increase in *CHOP* expression caused by the combination of DOX, LAP, and PTE-L was abolished by HIF1α inhibition (Figure 6B). HIF1α inhibition also suppressed apoptosis induced by the combination of anticancer drugs with PTE-L (Figure 6C). Furthermore, the inhibition of RNA-dependent protein kinase (PKR)-like ER kinase (PERK), an ER stress sensor related to the MAM, [16] also suppressed apoptosis induced by the combination of anticancer drugs with PTE-L (Figure 6D).

These results indicate that DOX, LAP, and PTE-L increased mtFe by suppressing the expression of PDZD8 and of the iron transporters recruited to PDZD8, i.e., mtNEET and ABCB8. Consequently, mitochondrial reactive oxygen species (ROS) levels increased, which increased HIF1α expression, which further induced ER stress as well as apoptosis.

### 2.6. Effect of Combination of PTE-L and Anticancer Drugs in Mouse Subcutaneous Tumor Model

**Figure 7:**
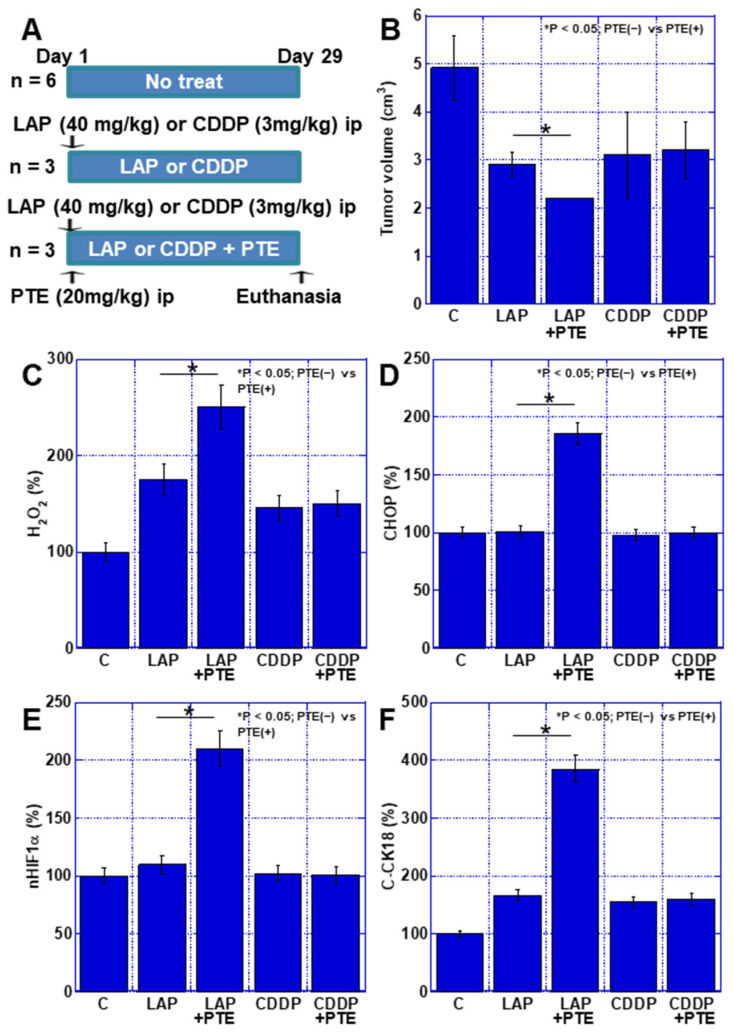
Effect of PTE on the antitumor effect of LAP and CDDP in a mouse tumor model. (**A**) Experimental protocol. TMK1 GC cells (1 × 10^7^) were inoculated subcutaneously in mice. On day 1, LAP (40 mg/kg, ip) or CDDP (3 mg/kg, ip), with or without PTE (20 mg/kg, ip), was administered. On day 29, mice were euthanized. (**B**) Tumor volume on day 29, which was calculated according to the formula (minor axis^2^ × major axis) / 2. * *p* < 0.05; PTE(−) vs. PTE(+). (**C**–**F**) Using whole-cell lysates of the tumors, the following parameters were measured using enzyme-linked immunosorbent assay: H_2_O_2_ (**C**), CHOP (**D**), nHIF1α (**E**), and C-CK18 (**F**). * *p* < 0.05; PTE(−) vs. PTE(+). Error bar, standard deviation determined by the number of mice constituting each group. * Significance was calculated using ANOVA with Bonferroni correction. GC, gastric cancer; PTE, pterostilbene; C, no treatment; LAP, lapatinib; CDDP, cisplatin; CHOP, C/EBP homologous protein; nHIF, nuclear hypoxia-inducible factor; C-CK18, cleaved cytokeratin 18.

Finally, the effect of the combination of PTE and anticancer drugs was examined in vivo. TMK1 cells were subcutaneously inoculated into nude mice and mtFe-depositing LAP or non-mtFe-depositing CDDP was administered (Figure 7A). The synergistic effect of PTE-L and LAP resulted in a greater reduction in tumor growth than that achieved with LAP alone, whereas the combination of CDDP and PTE-L did not have any effect on tumor growth (Figure 7B). When the levels of CHOP, H_2_O_2_, HIF1α, and cleaved cytokeratin 18 (C-CK18, an apoptosis marker) were measured in subcutaneous tumors, CHOP, HIF1α, and C-CK18 levels were all increased when the drug was used in combination with PTE-L, as compared to when LAP was used alone. In contrast, when CDDP and PTE-L were used in combination, no increase in these factors was observed (Figure 7C–F).

## 3. Discussion

In this study, we revealed that the combination of DOX or LAP with PTE-L inhibited the recruitment of the iron transporters mtNEET and ABCB8 to PDZD8, thereby causing the accumulation of mtFe and increasing mitochondrial ROS production. This resulted in HIF1α activation, which further induced ER stress and induced apoptosis.

In this study, PTE induced apoptosis in GC cells either when used alone at high concentrations (Figure 1) or when used at lower concentrations in combination with mtFe-accumulating anticancer drugs (Figure 4). The associated mechanisms included increased *BAX* and decreased *BCL2* expression [17,18,19], activation of c-Jun N-terminal kinase and p38 pathways [20,21], and further inhibition of the receptors for advanced glycation end-product/signal transducer and activator of transcription 3 and AKT/mechanistic target of rapamycin pathways [22]. In our study, a decrease in *BCL2* and an increase in *BAX* expression levels played important roles in PTE-H, whereas the combination of PTE-H and mtFe-depositing anticancer drugs did not induce ER stress-induced apoptosis.

We found that DOX, LAP, and PTE led to mtFe accumulation (Figure 2). DOX has been investigated as an anticancer drug known to accumulate mtFe. LAP is an anticancer drug used for HER2-positive GC, and 5FU and CDDP were investigated as anticancer drugs that are frequently used for GC. DOX results in the accumulation of mtFe and ROS production [13]. In contrast, the iron transporter ABCB8 reduces mtFe- and DOX-induced toxicity [13]. However, there have been no studies on the effects of LAP, 5FU, and CDDP on meFe accumulation.

SUN is a small molecule and a multitarget receptor tyrosine kinase inhibitor that mimics ATP and suppresses the phosphorylation of AKT and extracellular signal-regulated kinase (ERK), which are located downstream of the signal transduction pathway mediated by SUN, epithelial growth factor receptor, and HER2. We previously reported that SUN suppresses PDZD8 expression, leading to mtFe accumulation [12]. The original molecular targets and mechanisms of LAP, which inhibit intracellular signal transduction, are different from those of SUN. However, LAP, SUN, and DOX suppress PDZD8 as off-targets [12]. PDZD8 in the MAM anchors the ER and mitochondria bringing the two types of organelles in close proximity, facilitating material exchange between them [13]. Specifically, PDZD8 transfers calcium ions and lipids between the ER and mitochondria [23,24].

Our data showed that the iron–sulfur cluster transporter mtNEET [25,26] and iron transporter ABCB8 [27,28] were associated with PDZD8 (Figure 5). This suggested that both iron transporters bind to PDZD8 and are located within the MAM, transporting iron between the mitochondria and ER. *PDZD8* knockdown resulted in mtFe accumulation, similar to that observed with SUN, supporting the above considerations [12]. However, the mechanism through which these drugs suppress PDZD8 expression remains unclear and requires further investigation.

Our data showed that the combined use of DOX or LAP with PTE-L induced stronger mitochondrial ROS generation than did DOX or LAP alone (Figure 3). This was accompanied by increased expression of HIF1α. The combination of PTE-L with DOX or LAP resulted in synergistic mtFe accumulation in GC cell lines. However, iron chelation with DFO did not reverse the cell death rate. This suggested that the retention of mtFe induced mitochondrial ROS production. Furthermore, we observed no change in mitochondrial oxidized lipids (mt-4HNE) and GPX4, which plays an important role in mitochondrial redox and negates the involvement of ferroptosis. Our data showed that the combination of PTE-L with DOX or LAP induced ER stress. During ER stress, the calcium-enhanced ER release generates mitochondrial ROS that are associated with MAM dysfunction [29]. This suggested that the increase in mitochondrial ROS levels caused by PTE-L involved crosstalk with ER stress.

Mitochondrial ROS are known to lead to HIF1α accumulation and activation [30,31]. Mitochondrial ROS binds to the mitochondrial electron transport chain complex III and HIF1α and stabilizes HIF1α protein [32]. This is supported by the fact that the knockdown of Rieske’s iron–sulfur protein of mitochondrial complex III prevents HIF1α protein stabilization [33]. Consequently, the invasive ability of cancer cells was reduced [34]. The hydroxyl radicals (·OH) generated by the iron-dependent Fenton reaction, not only in the mitochondria but also in the perinuclear ER, promote *HIF1A* expression [35]. Thus, increased oxidative stress promotes the expression, stabilization, and activation of HIF1α through a mechanism different from that of hypoxia.

In this study, ER stress was induced by the combination of PTE-L and anticancer drugs that accumulated mtFe (Figure 2 and Figure 6). PTE alone is a potent inducer of ER stress [36] and causes ER stress through imbalances in redox homeostasis [37]. Overexpression of HIF1α increases the transcription factor activity of the unfolded protein response pathway and CHOP expression, leading to ER stress [38]. This suggests that the combined use of PTE and anticancer drugs that cause the accumulation of mtFe induces ER stress by increasing mitochondrial ROS generation due to the mtFe accumulation, thereby upregulating and activating HIF1α.

In our study, ER stress and apoptosis inhibitors inhibited cell death caused by the combined use of PTE-L, DOX, and LAP (Figure 4). Sustained ER stress caused apoptosis due to maladaptation to ER stress [38,39,40]. The mechanism may involve the fact that the ER stress sensor PERK aggregates at the MAM and mediates ROS signals between the ER and mitochondria [16]. The activation of PERK by unfolded proteins in the ER leads to the phosphorylation of the eukaryotic translation initiation factor 2α [41], which induces the activation of CHOP, GADD34, and ATF3 and promotes the transcription of the relevant target genes [42,43,44]. PERK, thus, mediates apoptotic signals from CHOP [45]. Our data also suggest that the inhibition of HIF1α and PERK suppresses apoptosis, leading to apoptosis due to ER stress (Figure 6).

LAP, which exhibited a synergistic effect with PTE-L in the present study, is a second-line treatment for HER2-positive GC [46]. Moreover, SUN, which has previously been reported to have a synergistic effect with PTE, has no indications for GC. The present study suggests that drugs that cause mtFe accumulation may exert antitumor effects when used in combination with PTE. This indicates that LAP and SUN may be more widely applicable when used in combination with PTE. Although the total dose of DOX is limited due to its cardiotoxicity [47], combining it with PTE makes it possible to obtain antitumor effects at lower doses. This may delay the onset of the side effects of DOX. 

In the future, by investigating the mtFe accumulation effect of anticancer drugs, it may be possible to identify drugs whose antitumor effect will be enhanced when combined with PTE.

## 4. Materials and Methods

### 4.1. Cell Lines and Reagents

Human gastric carcinoma cell lines TMK1 (poorly differentiated adenocarcinoma) and MKN74 (well-differentiated adenocarcinoma) were gifts from Professor Wataru Yasui (Molecular Pathology, Hiroshima University, Hiroshima, Japan) [48,49,50]. The human monocytic cell line U937 was purchased from Dainihon Pharmacy Co. (Tokyo, Japan). TMK1 and MKN74 cells were cultured in Dulbecco’s modified Eagle’s medium (Wako Pure Chemical Corporation, Osaka, Japan) supplemented with 10% fetal bovine serum (Sigma-Aldrich Chemical Co., St. Louis, MO, USA) at 37 °C in 5% CO_2_. 

LAP, DOX, 5FU, CDDP (Wako), and PTE (Tokyo Chemical Industry Co., Ltd., Tokyo, Japan), echinomycin (HIF1α inhibitor, Abcam, Cambridge, UK), and GSK2656157 (PERK inhibitor, Selleck, Houston, TX, USA) were purchased. The cells were incubated for 48 h. The IC_20_s of the anticancer drugs were as follows: DOX, 0.13 and 0.1 µM; 7.8 and 8.0 µM; 5FU, 30 and 55 µM; CDDP, 2.0 and 2.3 µM in TMK1 and MKN74, respectively.

### 4.2. Cell Growth and Apoptosis

Cell growth was assessed using the 3-(4,5-dimethylthiazol-2-yl)-5-(3-carboxymethoxyphenyl)-2-(4-sulfophenyl)-2H-tetrazolium (MTS)-based Celltiter 96 aqueous one-solution cell proliferation assay kit (Promega Corporation, Madison, WI, USA), as previously described [10]. Absorbance was measured at 490 nm on a Multiskan FC Microplate Photometer (Thermo Fisher Scientific, Waltham, MA, USA). 

### 4.3. Reverse Transcription–Polymerase Chain Reaction

Reverse transcription–polymerase chain reaction (RT-PCR) was performed with 0.5 µg total RNA extracted from the three cell lines using the RNeasy kit (Qiagen, Germantown, MD, USA) to assess human and murine mRNA expressions. The primer sets used are listed in Table 1 and were synthesized by Sigma Genosys (St. Louis, MO, USA). The PCR products were electrophoresed on a 2% agarose gel and were stained with ethidium bromide. *ACTB* mRNA was amplified as the internal control.

### 4.4. Mitochondrial Imaging

Mitochondrial function was examined using fluorescent probes. Cells were incubated with the probes for 30 min at 37 °C and then imaged using a BZ-X710 All-in-One fluorescence microscope (KEYENCE, Osaka, Japan). We used dihydrorhodamine 123 (10 μM, Sigma) to assess oxidative stress, mitoGreen (100 nM, PromoCell GmbH, Heidelberg, Germany) to assess mitochondrial volume, tetrathylrhodamine ethyl ester (200 nM, Sigma) to assess mitochondrial membrane potential, and mitoFerrogreen (20 nM, Dojindo, Kumamoto, Japan) to assess mtFe. 

### 4.5. Immunoblot Analysis

To prepare whole-cell lysates, cells were washed twice with cold phosphate-buffered saline (PBS), harvested, and lysed with RIPA buffer containing 0.1% sodium dodecyl sulfate (SDS) (Thermo Fisher) [51]. The Minute Cytoplasmic and Nuclear Extraction Kit (Invent Biotechnologies, Inc., Plymouth, MN, USA) was used to extract the nuclear and cytosolic fractions of the cells. Protein assays were performed using the Protein Assay Rapid Kit (Wako). Lysates (20 μg) were subjected to immunoblot analysis by using 12.5% SDS-polyacrylamide gel electrophoresis, followed by electrotransfer onto nitrocellulose membranes (Bio-Rad, Hercules, CA, USA). The membranes were incubated with primary antibodies and peroxidase-conjugated IgG secondary antibodies (MBL, Nagoya, Japan). Protein expression was assessed using primary antibodies (Table 1). β-actin antibody (Abcam, Cambridge, MA, USA) was used to assess protein loading. Immune complex binding was visualized using a CSA system (DAKO, Carpinteria, CA, USA). 

### 4.6. Immunoprecipitation

Immunoprecipitation was performed according to a previously described method [52]. The lysates were pre-cleaned in a lysis buffer containing protein A/G agarose (Santa Cruz Biotechnology) for 1 h at 4 °C and subsequently centrifuged. The supernatants were then incubated with a precipitation antibody against PDZD8 (Bios Inc., Woburn, MA, USA) and protein A/G agarose for 1.5 h at 4 °C. Precipitates were collected through centrifugation, washed three times with wash buffer, and solubilized in 4× Laemmli sample buffer (Bio-Rad) and 2-mercaptoethanol (Sigma). Immunoblotting was performed using antibodies against mitoNEET and ABCB8 (Table 1). VerBlot for IP Detection Reagent HRP (Abcam) was used as the secondary antibody.

### 4.7. Enzyme-Linked Immunosorbent Assay

Enzyme-linked immunosorbent assay (ELISA) kits were used to measure the concentrations of hydrogen peroxide, human CHOP, human HIF1α, 4HNE, human GPX4, and cleaved CK18 (Table 1). The assay was performed using whole-cell lysates according to the manufacturer’s instructions. A brief description is as follows. A total of 50 μL (10 μg) of protein sample was added to the wells and incubated at room temperature for 2 h; then, the solution was discarded. Each well was washed four times with the attached washing solution. A total of 100 μL of diluted detection antibody was added to the wells and incubated for 1 h at room temperature before the solution was discarded. After washing the wells four times, 100 μL of diluted horse radish peroxidase conjugate was added to each well and incubated for 30 min at room temperature. After discarding the solution, the wells were washed four times. A total of 100 μL of chromogenic substrate was added to each well and reacted for 30 min in the dark, and 100 μL of a stop solution was added. Immediately after stopping the reaction, the absorbance of each well was measured.

### 4.8. Animals

Four-week-old male BALB/c Slc-nu/nu mice were purchased from SLC Japan, Inc. (Shizuoka, Japan). The animals were maintained and subjected to experiments in accordance with the institutional guidelines approved by the Committee for Animal Experimentation of Nara Medical University and the current regulations and standards of the Ministry of Health, Labor, and Welfare of Japan (No. 13480, 18 May 2023 and No. 13480-1, 17 October 2023). 

### 4.9. Animal Tumor Models

Subcutaneous murine tumor models were established by inoculating cancer cells (TMK-1:1 × 10^7^ per mouse) into the subcutaneous tissues of nude mice on day 0. For each cell line, 18 mice were randomly divided into five groups: Control, LAP alone, and LAP + PTE. LAP (40 mg/kg body weight, diluted with 5% dimethyl sulfoxide), CDDP (3 mg/kg body weight, diluted with 5% dimethyl sulfoxide), and PTE (20 mg/kg body weight, diluted with 5% dimethyl sulfoxide) were administered intraperitoneally on day 1. Mice in the control group were injected with 100 µL of PBS (Wako) into the intraperitoneal cavity. The tumor diameter was measured using calipers over the skin of each mouse on each treatment day. The mice were sacrificed on day 29, and the tumor tissues were subjected to ELISA.

### 4.10. Statistical Analysis

Statistical significance was calculated using analysis of variance (ANOVA) with InStat software (version 3.1; GraphPad, Los Angeles, CA, USA). Correlations were tested using Pearson’s correlation coefficients. A two-sided *p* value of <0.05 was considered to indicate statistical significance. 

## Figures and Tables

**Table 1:** Primer sets, antibodies, and ELISA kits.

Primer Set			
Gene symbol	Gene bank ID	Forward primer (5′–3′)	Reverse primer (5′–3′)
BCL2	M13994.1	acgacaaccgggagatagtg	catcccagcctccgttatcc
BAX	L22473.1	catgaagacaggggcccttt	cttccagatggtgagcgagg
GADD45	M60974.1	ggaggaattctcggctggag	tccatgtagcgactttcccg
CHOP	NM_001195053.1	ccagccactccccattatcc	ttcggtcaatcagagctcgg
PDZD8	NM_173791.4	tcctcgtgttgatgctgaag	ttgtctgacgtgttgggtgt
mitoNEET (CISD1)	BC007043.1	tccagaaagacaaccccaag	gcccacattgtctccagtct
ABCB8	NM_001282291.2	cgtggggtctcgctttaact	cctgacactggcgagacaat
HIF1α	AF208487.1	gaaagcgcaagtcctcaaag	tgggtaggagatggagatgc
PERK	NM_004836.7	gcagaggcagtggagtttct	ggcaaagggctatgggagtt
ACTB	NM_001101.3	ggacttcgagcaagagatgg	agcactgtgttggcgtacag
Antibody			
Protein	Clone	Company	
PARP	-	GeneTex, Irvine, CA, USA	
mitoNEET	L70G2	Biocompare, South San Francisco, CA, USA	
ABCB8	F-4	Santa Cruz, Santa Cruz, CA, USA	
PDZD8	-	Bioss Inc, Woburn, MA, USA	
β-actin	-	Abcam, Cambridge, MA, USA	
ELISA			
Items	Catalog number	Company	
Hydrogen peroxide	21024	Aoxre Bioscience, Burlingane, CA, USA	
Human CHOP	LS-F8872	Lsbio, Shirley, MA, USA	
Human HIF1α	EHIF1A	Thermo Fisher, Tokyo, Japan	
4HNE	ab238538	Abcam, Cambridge, MA, USA	
Human GPX4	ARP-E4145	Biocompare. South San Francisco, CA, USA	
Cleaved CK18, M30	10011	VLVbio, Nacka, Sweden	

BCL2, B cell lymphoma 2; BAX, Bcl-2-associated X protein; GADD45, growth arrest and DNA damage inducible 45; CHOP, CCAAT/enhancer-binding protein homologous protein; mitoNEET, mitochondrial protein containing Asn–Glu–Glu–Thr (NEET) sequence; ABCB8, ATP-binding cassette subfamily B member 8; PDZD8, PDZ domain-containing 8; PARP, poly ADP-ribose polymerase; HIF, hypoxia-inducible factor; HNE4, mitochondrial hydroxynonenal-4; GPX4, glutathione peroxidase 4; CK18, cytokeratin 18.

## Data Availability

Data are contained within the article.

## Abbreviation

GC: gastric cancer; PTE, pterostilbene; mtFe, mitochondrial iron (II); 5FU, 5-fluorouracil; CDDP, Cisplatin; DOX, doxorubicin; LAP, lapatinib; MAM, mitochondria-associated endoplasmic reticulum membrane; PDZD8, PDZ domain-containing 8; HER2, human epidermal growth factor receptor 2; SUN, sunitinib; ER, endoplasmic reticulum; FER, ferrostatin; DFO, deferoxamine; NAC, N-acetylcysteine; 4PBA, 4-phenylbutyric acid; PARP, poly[ADP-ribose] polymerase; GADD45, DNA damage-inducible; CHOP, CCAAT/enhancer-binding protein homologous protein; mitoNEET, mitochondrial protein containing the Asn-Glu-Glu-Thr (NEET) sequence; ABCB8, ATP-binding cassette subfamily B member 8; GPX, glutathione peroxidase; HIF, hypoxia-inducible factor; PERK, RNA-dependent protein kinase (PKR)-like ER kinase; ROS, reactive oxygen species; C-CK18, cleaved cytokeratin 18.
